# Rheological Properties of Gel Foam Co-Stabilized with Nanoparticles, Xanthan Gum, and Multiple Surfactants

**DOI:** 10.3390/gels9070534

**Published:** 2023-06-30

**Authors:** Youjie Sheng, Hanling Zhang, Li Ma, Zhenping Wang, Die Hu, Shanwen Zhang

**Affiliations:** College of Safety Science and Engineering, Xi’an University of Science and Technology, Xi’an 710054, China; zhanghanling920@163.com (H.Z.); wzp147258369@sina.com (Z.W.); hudie_0313@163.com (D.H.); 18615962035@163.com (S.Z.)

**Keywords:** gel foam, xanthan gum, nanoparticle, foam rheology, foam drainage

## Abstract

Gel foam has the advantages of gel and foam and shows good prospects for applications in the fields of fire prevention and extinguishing. Rheology has a significant impact on the application of gel foam, but there is little related research. In the present study, hydrophilic silica nanoparticles (NPs) and water-soluble polymer xanthan gum (XG) were combined with fluorocarbon surfactant (FS-50) and hydrocarbon surfactant (APG0810) to create gel foam. The foaming ability and foam drainage were evaluated. The gel foam’s rheology, including its flow behavior and viscoelasticity, was systematically investigated. The results show that the foaming of the FS-50/APG0810 mixture decreases but the foam drainage increases in the presence of NPs and/or XG. All of the foams belong to the category of non-Newtonian fluids with shear thinning behavior. The flow curves of the foams are consistent with the Cross model. The presence of XG/NPs enhanced the foam viscoelasticity of the FS-50/APG0810 mixture. The silica NPs showed a better ability to enhance foam viscoelasticity but a worse ability to stabilize the foam compared to XG. This research can offer theoretical support for the industrial usage of gel foam.

## 1. Introduction

Foam is a two-phase system made up of gas distributed in a continuous liquid phase, where the liquid part mostly consists of surfactant and the gas phase is composed of air. Foams have been used extensively in the food, mineral flotation, cosmetic, firefighting, and oil recovery industries [1,2,3,4,5,6,7]. However, foams are not suitable for many practical and industrial uses due to their thermodynamic instability and rapid collapse. Therefore, additional foam stabilizers need to be found to meet the actual demand.

Traditionally, foam stabilizers include water-soluble polymers like polyacrylamide and xanthan gum (XG), which have been researched extensively in the food, coal mining, cosmetic, and oil recovery industries [8,9,10,11]. XG has demonstrated its effectiveness as a foam stabilizer by increasing the viscosity of aqueous solutions in the field of firefighting foam [12]. Previous studies have shown that nanoparticles (NPs) can offer significant enhancement of the foam stability of surfactant solutions, including SiO_2_, Al(OH)_3_, CaCO_3_, and so on [7,13,14]. The improvement mechanism of NPs for foam stability is such that NPs can slow down foam drainage and retard gas transfer between bubbles through dispersal into bubble liquid films and Plateau borders [15,16,17]. A previous study showed that appropriate concentrations of SiO_2_ NPs can dramatically improve foam stability in foaming liquids containing fluorocarbon and hydrocarbon surfactants [6]. Moreover, previous studies have shown that adding polymers and NPs into surfactant systems enhances foam stability in a synergistic manner [5]. However, previous studies have mainly discussed the effects of NPs or XG on the static properties of firefighting foam. The rheological properties of the foam, such as elasticity, plasticity, and viscosity, are critical to the application of foam in systems and equipment [18,19]. During the fire extinguishing process, the internal structure of the foam is changed dynamically from the foam’s injection to the complete coverage of the fuel surface. Therefore, the foam’s flow behavior and viscoelasticity under external shear tend to affect the fire extinguishing performance to a certain extent. Hence, the rheological properties of foam synergistically stabilized with polymer and NPs warrant investigation.

In this study, a gel foam was created based on hydrophilic silica NPs, water-soluble polymer XG, hydrocarbon surfactant, and fluorocarbon surfactant. The effects of different foam stabilizers on the properties of the gel foam were systematically investigated, including the foaming ability, foam stability, and foam rheological properties. It is then thoroughly explained how XG and NPs work together to improve foam stability, foam flow behavior, and foam viscoelasticity. The results can provide a foundation for the industrial usage of new forms of gel foam in firefighting.

## 2. Results and Discussion

### 2.1. Characterization of the Mixed Dispersions

**Figure 1:**
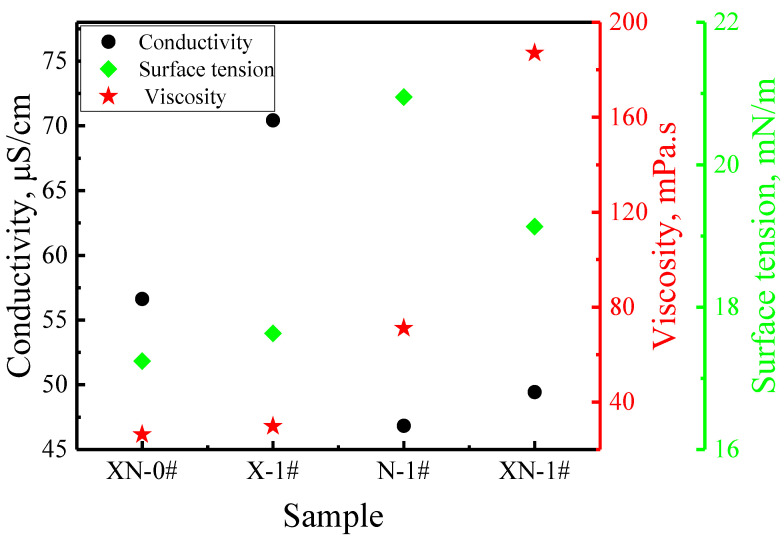
Basic properties of the mixed dispersions with different components.

The basic properties of the mixed dispersions are presented in Figure 1. The surface tension of the FS-50/APG0810 dispersion in the absence of XG and NPs is 17.24 mN/m. A previous study indicated that the surface tensions of individual solutions of APG0810 and FS-50 are 26.89 mN/m and 15.93 mN/m, respectively [20]. The value of 17.24 mN/m is between 26.89 mN/m and 15.93 mN/m, indicating the coexistence of FS-50 and APG0810 molecules at the gas–liquid interface within the surfactant mixture. The surface tension of the FS-50/APG0810 dispersion containing XG increased slightly, but the surface tension of the FS-50/APG0810 dispersion containing NPs increased significantly, indicating that the NPs exerted a greater effect on molecular interactions at the gas–liquid interface than XG. Adding NPs to the FS-50/APG0810/XG dispersion led to an increase in surface tension, implying that the presence of NPs disrupts surfactant adsorption at the gas–liquid interface. Previous studies indicated that SiO_2_ NPs are negatively charged in water [21], and XG is an anionic polymer with a negative charge in water [22]. FS-50 is amphoteric and APG0810 is nonionic. Hence, Coulomb forces exist among molecules with the same charge in the mixed dispersion of FS-50/APG0810/XG/NPs. At the same time, the negatively charged XG/NPs and the FS-50 molecules with amphoteric ions exhibit attractive forces. It should be noted that an adsorption interaction exists between NPs and surfactant molecules. The nonionic APG0810 molecules can adsorb onto the surface of NPs through van der Waals forces, leading to the formation of aggregates [23], and strong adsorption between SiO_2_ NPs and the amphoteric FS-50 molecules is generated through hydrogen bonding [24,25]. The adsorption of FS-50 on the surface of SiO_2_ NPs results in an obvious reduction in the number of FS-50 molecules at the gas/liquid interface, the transformation of NPs from hydrophilic particles to hydrophobic aggregates, and a corresponding increase in surface tension.

The conductivity of the FS-50/APG0810 dispersion increases with the addition of XG, while the conductivity of the dispersion decreases with the addition of NPs. The conductivity of the FS-50/APG0810/XG/NPs dispersion is between that of the FS-50/APG0810/XG and that of the FS-50/APG0810/NPs. The main reason for this phenomenon is that the addition of XG generates more free charges in the foam dispersions due to its larger relative molecular mass compared to that of NPs. In addition, the adsorption of APG0810 molecules on the negatively charged SiO_2_ NPs decreases the free charge and the corresponding conductivity of the FS-50/APG0810 solution.

The inclusion of XG and NPs increases the viscosity of the FS-50/APG0810 dispersion, but the viscosity of the FS-50/APG0810/NPs dispersion is higher than that of the FS-50/APG0810/XG. The main reason for this is the high NP concentration and the formation of considerable aggregates owing to the adsorption of surfactant molecules on the NP surface. The viscosity of the mixed dispersion is greater than that of the other dispersions in the simultaneous presence of NPs and XG, up to 187.11 mPa.s, indicating that the interactions between the surfactants, XG, and NPs produced a cooperative thickening influence. These results show that NPs have stronger interactions with FS-50/APG0810 molecules than XG, and that the complex and strong molecular interactions lead to changes in surface tension, viscosity, and conductivity.

### 2.2. Foam Properties

#### 2.2.1. Foaming Ability

**Figure 2:**
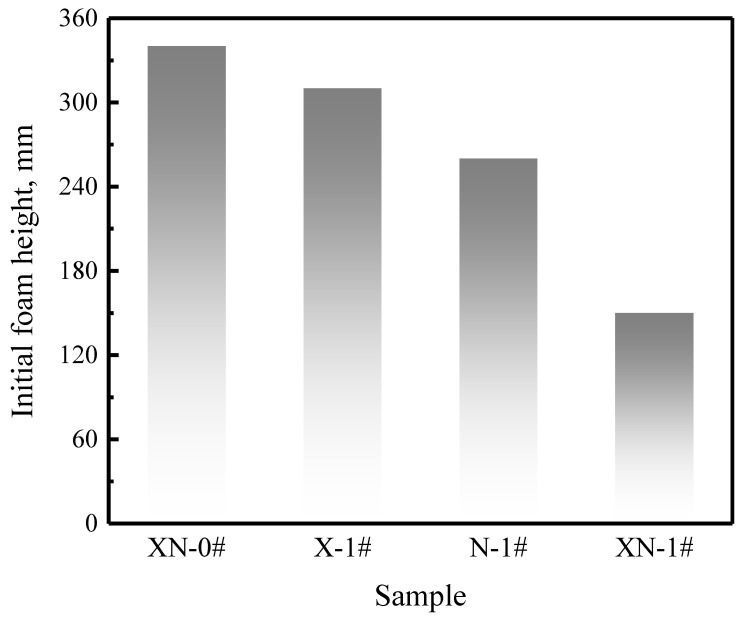
Initial foam height of mixed dispersions with different components.

Figure 2 illustrates the initial foam height of the mixed dispersions. Adding 5% NPs and 0.05% XG to the FS-50/APG0810 dispersion reduces the initial foam height. The initial foam height of FS-50/APG0810/NPs/XG is dramatically decreased by adding XG to the FS-50/APG0810/NP dispersion. Notably, FS-50/APG0810/XG has a higher foaming ability than the FS-50/APG0810/NPs. The foaming ability of surfactant solutions is a complex process. It is influenced by various factors, such as surface tension, viscosity, conductivity, particle aggregation, and so forth. Prior research has demonstrated that an increase in viscosity hinders the creation of bubbles, resulting in a reduced foaming ability [26,27]. In the current study, an increase in dispersion viscosity was the primary cause contributing to the lower foam height.

#### 2.2.2. Foam Drainage

**Figure 3:**
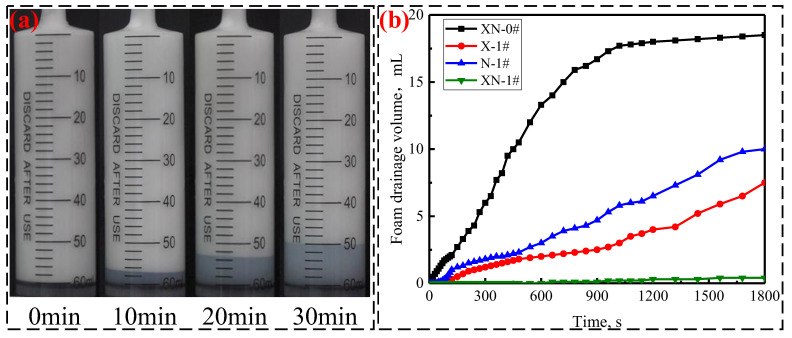
Foam drainage of foams containing different components: (**a**) foam drainage process over time for N-1# and (**b**) foam drainage volume.

Figure 3 illustrates the foam drainage of foams containing different components. As depicted in Figure 3a, the foam volume in the syringe decreased slowly with time, while the volume of liquid drained increased. However, the upper surface of all the foams was not altered after 30 min. As depicted in Figure 3b, all of the drainage curves of the four foams increased over time. The drainage curves of the other three foams are lower than that of the FS-50/APG-0810 foam (XN-0#), implying that XN-0# exhibits more rapid drainage than the other three foams. Notably, the drainage curve of XG stabilized foam (X-1#) is lower than that of NPs stabilized foam (N-1#), indicating that the stabilization ability of XG for FS-50/APG0810 foam is better than that of NPs. The drainage curve of XG and NPs collectively stabilized foam (XN-1#) is lower than that of the XG-stabilized foam (X-1#) and NP-stabilized foam (N-1#), demonstrating the synergistic impact of the XG and NPs on the foam stability of APG0810/FS-50.

Many factors have been proposed to explain the effect mechanism of NPs and/or XG on foam stability, including surface activity, viscosity, particle aggregation at the liquid films and Plateau borders, the maximum capillary pressure of coalescence, and gas diffusion between bubbles [21,28,29,30,31]. Previous studies have shown that NPs at a suitable concentration can retard foam drainage by forming a stable meshwork structure in foam films and Plateau borders [32,33]. In the present study, the mechanism of NP-stabilized foam can also be explained by the interaction between NPs and surfactant molecules and the formation of aggregates formed through their interaction in the liquid films and Plateau borders. Moreover, according to a prior study, a gel-like membrane forms in the foam due to the interaction or cross-linking between XG molecules [34]. It is the presence of the gel film that prevented foam drainage in the present study. As previously stated, Coulombic forces are present in mixed dispersions of FS-50/APG0810/XG/NPs. Coulombic repulsive interactions between identically charged molecules of FS-50/APG0810/XG/NPs enhance the aggregation of NPs at the foam films and Plateau borders and the interaction or cross-linking between XG molecules. Hence, the aggregates of NPs in the foam films and Plateau borders and the gel-like film formed through the interaction or cross-linking of XG molecules jointly delay foam drainage, leading to the improvement of foam stability.

### 2.3. Foam Rheology

#### 2.3.1. Flow Behavior

**Figure 4:**
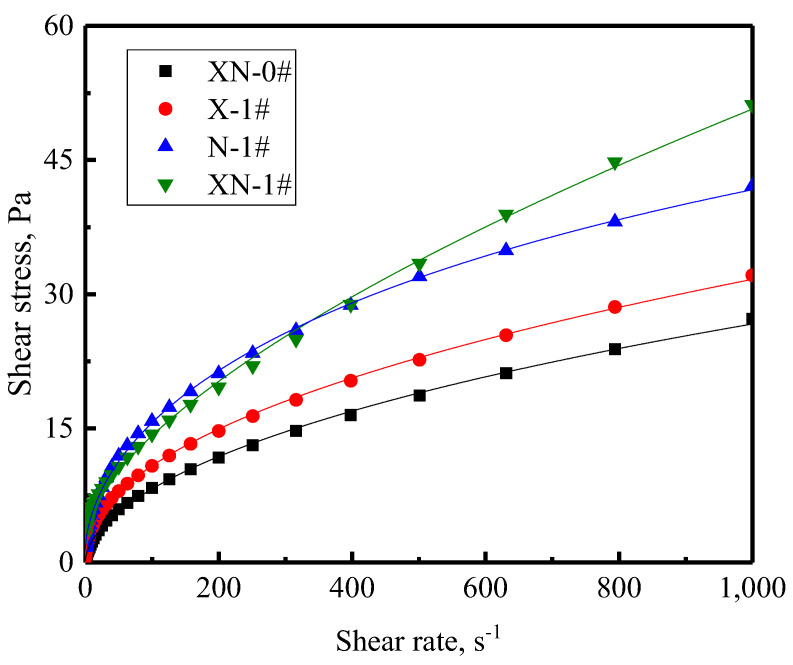
Foam shear stress variation over the shearing rate.

Figure 4 illustrates the variation curves of shear stress on the foam versus the shear rate. The shear stress on the four foams showed a nonlinear variation with the shear rate, indicating that the four foams exhibited the rheological behavior of non-Newtonian fluid. At the same shear rate, the shear stress on the foams with the presence of XG and/or NPs gradually increases, implying that the shear stress required to cause the deformation of foams stabilized using XG and/or NPs is greater than that of foams without them.

Table 1 shows the results of the model parameters fitted to the flow curves of different foams using the Cross model. The data shown in Table 1 show that the τ_0_ of the foam, which was merely stabilized by the surfactant, is 0 Pa, indicating that XN-0# is a pseudoplastic fluid. The τ_0_ of the other three foams gradually increases with the inclusion of XG and/or NPs, demonstrating that the foams are pseudoplastic fluids and have higher yield strengths than those stabilized with surfactants. In addition, the correlation coefficients R^2^ of all the foams are very close to 1, indicating that the Cross model can fit the flow curves of all of the foams well.

**Figure 5:**
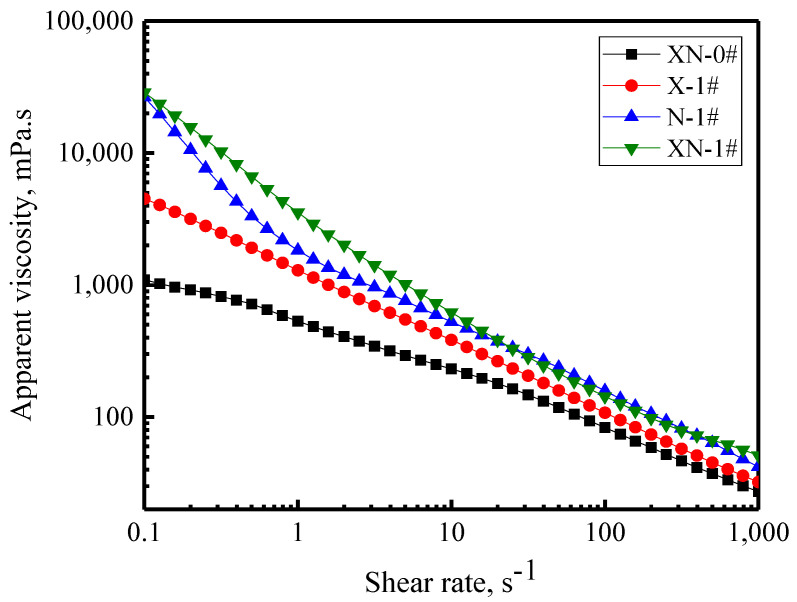
Foam apparent viscosity variation over the shear rate.

Figure 5 depicts the variation in apparent viscosity of the four foams over the shear rate. The apparent viscosity of the four foams reduces as the shear rate increases, further demonstrating that the four foams are non-Newtonian fluids with shear thinning behavior [35]. The reason for this behavior is that the bubbles and the aggregates formed by the foam components in the bubbles are destroyed by the shear force, caused by the gradually increasing shear rate, resulting in a reduction in foam viscosity [36]. For the foams without XG or NPs, the aggregates should be micelles formed by FS-50/APG0810 molecules. For the foams with XG, the aggregates should be micelles formed by FS-50/APG0810 molecules due to cross-linking and entanglement between the surfactant molecules and XG molecules [37]. For the foams with NPs, the aggregates should be created through the adsorption of FS-50 molecules and APG0810 molecules on the surface of NPs via hydrogen bonding and van der Waals forces. For the foams with XG and NPs, the aggregates should be created by FS-50 molecules, APG0810 molecules, XG molecules, and NPs (the silanol group on the surface of SiO_2_ NPs interacts with the carboxyl group (COOH) on the side chain of XG trisaccharide through hydrogen bonding [38]). With the inclusion of XG and/or NPs, the foam flow behaviors of the FS-50/APG0810 dispersions are unchanged, but the value of the foam’s apparent viscosity at the same shear rate significantly increases. This can be attributed to the fact that the incorporation of XG increases the viscosity of the dispersion, which in turn increases the apparent viscosity of the foam. In addition, the aggregates created by the FS-50 molecules, APG0810 molecules, and NPs stopped the shear process with the addition of NPs, resulting in the apparent viscosity being increased. These findings show that the shear rate necessary for a total breakdown of the foam structure gradually increases with the inclusion of XG and/or NPs. In comparison to the micelles generated by FS-50/APG0810 molecules and the aggregates created by surfactants and XG, the aggregates generated by surfactants and NPs show greater resistance to external damage and, to some extent, slow down the shear thinning process.

#### 2.3.2. Viscoelasticity

**Figure 6:**
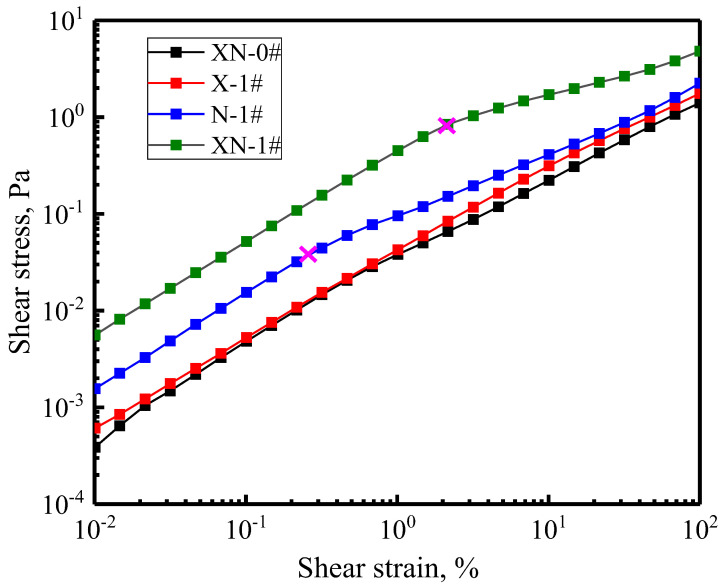
Variation in the shear stress of foam versus the shear strain.

Figure 6 depicts the variation curves of shear stress on the foam versus shear strain. The shear stress for all the foams increases as the shear strain increases. Specifically, the stress–strain curves of N-1# and XN-1# have an obvious inflection point (shown by the pink arrow). The inflection point is defined as the foam yield point, as used in a previous study [39]. Irreversible deformation of the foam structure occurred after the yield point. The shear stress values at the inflection points of the N-1# and XN-1# curves are approximately 0.04 and 0.8 Pa, respectively. These results indicate that the foam yield limit of FS-50/APG0810 is significantly increased in the presence of both XG and NPs. Notably, the stress–strain curves of XN-0# and X-1# almost overlap with each other, and no yield point occurs on the two curves. Upon the addition of NPs, the shear stress on the foam increases. These findings demonstrate that the foams containing NPs show stronger resistance to deformation than those without NPs, although XG exhibits a stronger ability to improve foam stability than NPs.

**Figure 7:**
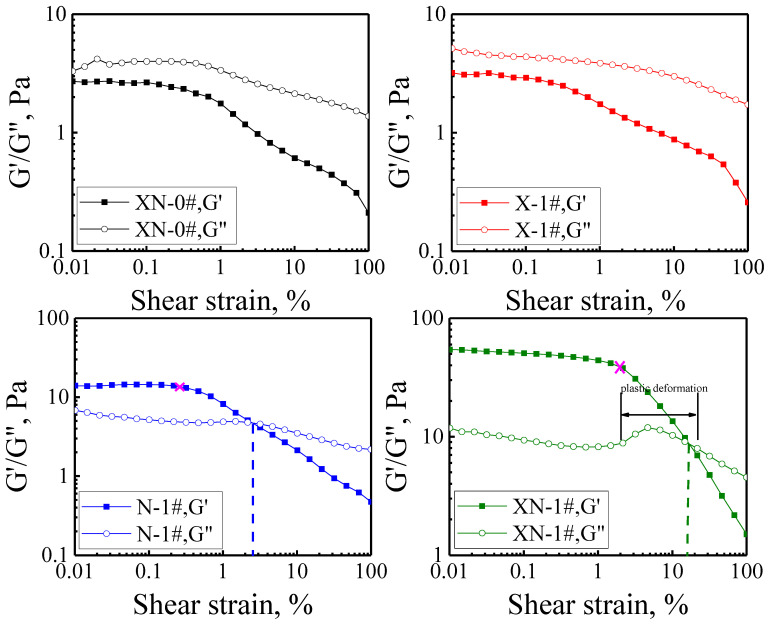
Variation in the viscoelastic modulus of foam over shear strain.

Figure 7 illustrates the variation in the viscoelastic modulus of the foams over the shear strain. For all the foams, the G’ curves essentially remain unchanged for reasonably small shear strains but decrease quickly above a specific shear strain. The inflection points on the G’ curves can be considered as the yield point of the foams [40]. The reason for this phenomenon is that the applied shear strain is sufficient to separate the adjacent bubbles, the foam structure yields and deforms, and the bubbles slide against each other after the yield point [36]. The G” curve decreases gradually with increasing strain. It is worth noting that the G” curve for XN-1# appears to rise briefly and then fall at high shear strains (area in black). Prior research suggested that this phenomenon is associated with the plastic deformation of the foam before yielding [41]. The G’ values of XN-0#, X-1#, N-1#, and XN-1# are 2.7, 3.2, 13.9, and 54.2 Pa, respectively, at low strain. These results demonstrate that the viscoelasticity of NP-enhanced foam is greater than that of XG-enhanced foam. The reason is that the FS-50/APG0810/NPs/XG aggregates filling the foam film and Plateau borders strengthen the structure of the foam film, which in turn improves the ability of the foam to resist shear strain.

For XN-0# and X-1#, the viscoelastic response behavior remains viscous throughout the strain range due to G” > G’, further indicating that no yield point occurs on the curves of XN-0# and X-1# at the strain range of 0.01–100%. Notably, a point where G’ = G” appears in the modulus curves of XN-1# and N-1#, and the point is called a flow point [40]. The flow point reacts to the foam fluid changing from a gel-like solid to a fluid-like liquid [41,42]. The value of G’ is greater than that of G” before the flow point, where the viscoelastic response behavior is dominated by elasticity. However, the value of G’ is smaller than that of G” after the flow point, where the viscoelastic response behavior is dominated by viscidity. The values of shear strain corresponding to the flow points for N-1# and XN-1# are 3.18% and 14.8%, respectively. This result indicates that XG and NPs interact with one another to improve the foam viscoelasticity of FS-50/APG0810.

## 3. Conclusions

A comprehensive experimental investigation was conducted to examine the effects of XG and NPs on foam rheology and stability. Foaming ability, foam stability, and foam rheological properties are significantly influenced by the addition of NPs and water-soluble polymer as foam stabilizers. The primary conclusions are as follows.

Stronger molecular interactions exist in FS-50/APG0810/XG/NPs dispersions. The presence of XG and/or NPs results in an increase in the surface tension and viscosity of the FS-50/APG0810 mixture. The conductivity of the FS-50/APG0810 mixture increases significantly with the addition of XG but decreases significantly with the addition of NPs.

The foaming ability of the FS-50/APG0810 dispersion is diminished with the presence of NPs and/or XG. The presence of XG and/or NPs can effectively retard foam drainage and thus improve the foam stability of the FS-50/APG0810 dispersion. In terms of the present foam components, XG exhibits a stronger ability to improve foam stability than NPs.

All of the foams used in the present study are non-Newtonian fluids with shear thinning behavior, and their flow curves are consistent with the Cross model. The presence of NPs significantly enhances the foam yield limit. The presence of XG and/or NPs improves foam viscoelasticity, but the NP-stabilized foam exhibits greater viscoelasticity than that stabilized with XG. The viscoelastic response behavior of foams containing NPs exhibits an elastic response, but foams without NPs exhibit a viscous response at low strain values. Large amounts of aggregates produced by FS-50/APG0810/NPs/XG are disseminated into the foam films and Plateau borders, which are the primary contributors to the improved foam yield limit and viscoelasticity.

## 4. Experimental

### 4.1. Materials

**Figure 8:**
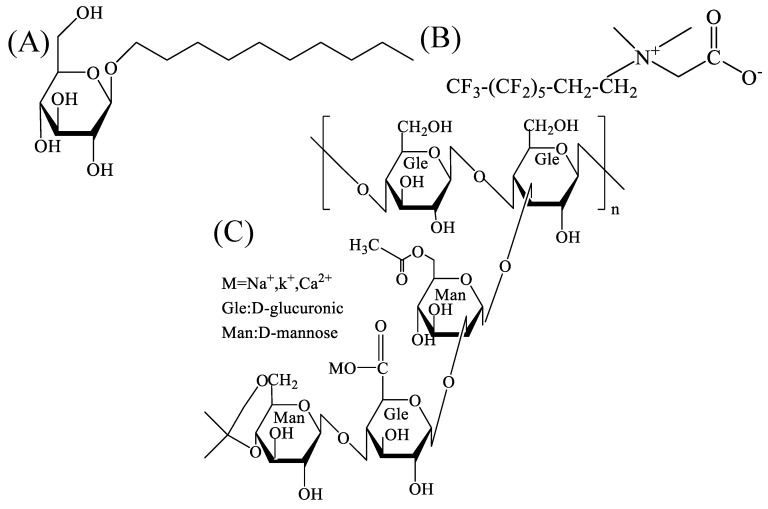
Molecular formula: (**A**) APG0810, (**B**) FS-50, and (**C**) xanthan gum (XG).

Hydrophilic gas-phase SiO_2_ NPs (99.8 wt%) were acquired from Shanghai Aladdin Bio-Chemical Technology Co., Ltd. (Shanghai, China). Their average specific surface area was 300 m^2^/g. The nonionic hydrocarbon surfactant alkyl glucoside APG-0810 (50%), with the molecular structure as shown in Figure 8A (the rest of the ingredients were water and alcohols), was obtained from Shandong Yousuo Chemical Technology Co., Ltd. (Linyi, China). The C6 fluorocarbon surfactant with a zwitterion, Capstone^®^ FS-50, was procured from DuPont; 27% of the FS-50 contained active components, and the others were water and a little ethanol. Its molecular structure, provided by the manufacturer, is shown in Figure 8B. Xanthan gum (XG), with the molecular structure shown in Figure 8C and a purity of 99.9%, was obtained from Tianjin Bailunsi Biotechnology Co., Ltd. (Tianjin, China).

### 4.2. Apparatus and Methods

#### 4.2.1. Generation Testing of Mixed Dispersions

Table 2 lists each component of the mixed dispersions used in the current study and the associated concentrations. Previous studies showed that mixed dispersions with 0.05% XG or 5% NPs still exhibited excellent foam stability as well as good foaming ability [6,37]. Hence, in the present study, the concentrations of the XG and NPs were fixed at 0.05% and 5%, respectively. The concentrations of FS-50 and APG0810 were above their critical micelle concentrations (CMCs), 0.0126 wt% and 0.25 wt%, as obtained from our previous studies [43,44]. The surface tension of the mixed dispersions was measured using a QBZY-3 automatic surface tension meter based on a hanging piece method. During the test, a hanging piece (platinum plate) was lowered gradually towards the mixed dispersion. The platinum plate was stopped once the lower boundary of the platinum plate touched the upper surface of the mixed dispersion. The surface tension value of the mixed dispersion was outputted from the surface tension meter. A prior study provides a thorough description of the test procedures [6]. The conductivity of the mixed dispersion was characterized using a SG23-B Mettler multiparameter tester. During testing, two parallel electrode plates were put into the measured mixed dispersion. The current flowing between the plates was measured after adding a certain potential at both ends of the plates. The conductivity value of the mixed dispersion was then outputted from the multiparameter tester. The MCR302e rheometer developed by Anton Paar was used to characterize the rheological properties of the mixed dispersions. During testing, the coaxial cylinder system was chosen to test the viscosity of the mixed dispersions. For this, 4 mL dispersions were added to a standard vessel with a diameter of 26.7 mm, and the test temperature and shear rate were set to 25 °C and 0.1 s^−1^, respectively. The viscosity values were obtained after running the program for a few minutes. For all of the dispersions, each test was conducted at least three times in order to obtain an accurate value.

#### 4.2.2. Characterization of Foam Rheology and Foam Properties

##### Foam Rheology

Dynamic rheology is an important parameter that determines the viscous and elastic properties of foam. The constitutive equation of fluid is a mathematical model used to describe the relationship between the fluid shear stress and shear rate [45]. The constitutive equation has great significance for the in-depth study of rheological properties and practical engineering field applications [46]. Hence, in the present study, we fitted the flow curve to the Cross model shown in Equation (1) [47,48]:

(1) $$\tau =\frac{\tau_{0}-\tau_{\infty }}{1+{\left(\lambda \gamma \right)}^{m}}+\tau_{\infty }$$

where *τ* is shear stress, Pa; *τ*_0_ is shear stress as the shear rate approaches zero, Pa; *τ*_∞_ is shear stress as the shear rate approaches infinity, Pa; *λ* is the characteristic time and related to the value of the shear rate at the beginning of the shear thinning behavior, s; *γ* is the shear rate, s^−1^; and *m* is the non-Newtonian index. An MCR302e rheometer produced by Anton Paar was used to analyze the rheological response of the foam. The rheological experiments were carried out by utilizing a PP50 rotor and plate test equipment. The test interval was set to 0.1 mm and the temperature was fixed at 25 °C. The shear rate variation was fixed within the range of 0.1 s^−1^ to 1000 s^−1^ for evaluating the foam’s steady-state rheological behavior. An amplitude sweep test was conducted for the foam at a constant temperature. The viscoelasticity of foam was measured at a constant frequency (10 rad s^−1^) and a strain amplitude ranging from 0.1% to 100%.

##### Foaming Ability and Foam Drainage

Foam properties mainly include foaming ability and foam drainage. Foaming ability is one of the most essential properties of foam materials. In the present study, a modified Ross–Miles approach that has been commercially successful was used to test foaming ability. A previous work contains a detailed description of the experimental setup and test method [6]. The initial foam height was used to assess foaming ability. The double-syringe method was employed to create a foam and evaluate the foam drainage, as described in detail in a previous study [6]. The foam drainage procedure was captured through a CCD camera.

## Figures and Tables

**Table 1:** Simulation parameters of Cross model.

Sample	Model Parameter				
	τ_0_ (Pa)	τ_∞_ (Pa)	λ (s)	m	R^2^
XN-0#	0	205.15	3.24 × 10^−5^	0.56	0.9993
X-1#	0.15	2582.29	9.69 × 10^−8^	0.48	0.9997
N-1#	1.17	119.57	3.19 × 10^−4^	0.57	0.9985
XN-1#	3.22	9214.19	2.67 × 10^−7^	0.64	0.9986

**Table 2:** Mixed dispersions with different components.

Sample	FS-50(%)	APG0810(%)	XG(%)	NP(%)
XN-0#	0.25	0.5	0	0
X-1#	0.25	0.5	0.05	0
N-1#	0.25	0.5	0	5
XN-1#	0.25	0.5	0.05	5

## Data Availability

Data is contained within the article.
